# Polypharmacy and Pharmacological Treatment of Diabetes in Older Individuals: A Population-Based Study in Quebec, Canada

**DOI:** 10.3390/pharmacy7040161

**Published:** 2019-12-01

**Authors:** Marie-Eve Gagnon, Caroline Sirois, Marc Simard, Céline Plante

**Affiliations:** 1 Department of Social and Preventive Medicine, Université Laval, 1050 Avenue de la Médecine, Québec, QC G1V 0A6, Canada; marie-eve.gagnon.33@ulaval.ca; 2 Centre d’Excellence sur le Vieillissement de Québec, 1050, Chemin Sainte-Foy, Québec, QC G1S 4L8, Canada; 3 Department, Institut National de Santé Publique du Québec, 945 Avenue Wolfe, Québec, QC G1V 5B3, Canada; marc.simard@inspq.qc.ca (M.S.); celine.plante@inspq.qc.ca (C.P.)

**Keywords:** medication, diabetes, aged, pharmacoepidemiology, polypharmacy, population-based cohort

## Abstract

Our objectives were to describe the use of pharmacological treatments in older adults with diabetes and to identify the factors associated with the use of a combination of hypoglycemic, antihypertensive and lipid-lowering agents. Using the Quebec Integrated Chronic Disease Surveillance System, we conducted a population-based cohort study among individuals aged 66–75 years with diabetes in 2014–2015. We described the number of medications and the classes of medications used and calculated the proportion of individuals using at least one medication from each of these classes: hypoglycemics, antihypertensives and lipid-lowering agents. We identified the factors associated with the use of this combination of treatments by performing robust Poisson regressions. The 146,710 individuals used an average of 12 (SD 7) different medications, mostly cardiovascular (91.3% of users), hormones, including hypoglycemic agents (84.5%), and central nervous system medications (79.8%). The majority of individuals (59%) were exposed to the combination of treatments and the factor most strongly associated was the presence of cardiovascular comorbidities (RR: 1.29; 99% CI: 1.28–1.31). Older individuals with diabetes are exposed to a large number of medications. While the use of the combination of treatments is significant and could translate into cardiovascular benefits at the population level, the potential risk associated with polypharmacy needs to be documented.

## 1. Introduction

With the aging of the population, the prevalence of chronic diseases such as diabetes is expected to increase sharply [1]. In Canada, the proportion of individuals with diabetes should rise from 9.3% in 2015 to 12.1% in 2025 [2]. Furthermore, 54.8% of older individuals with diabetes have three or more other chronic comorbidities [3], which adds to the burden of disease and medications.

Clinical practice guidelines are useful tools to support health care decisions. In Canada, diabetes practice guidelines recommend antidiabetic agents (including insulin) and cardioprotective therapies (antihypertensives, notably ACE inhibitors and angiotensin-receptor blockers (ARB), and lipid-lowering agents, notably statins) for individuals aged ≥55 with diabetes. Additionally, antiplatelet agents are recommended for individuals with established cardiovascular disease, mainly for secondary prevention [4]. 

Currently, there is limited information on the overall medication profile of older individuals with diabetes. Little is known about the proportion of individuals who are exposed to a combination of antidiabetic agents and cardioprotective therapies, and on the factors that are associated with such a combination. Studies have shown that medication use, in general, is higher in individuals aged ≥65 and among women [5,6]. And yet, women are less likely to receive pharmacological treatment consistent with diabetes and cardiovascular guidelines [6], which may result from the historical underestimation of the cardiovascular risk among women and lack of referral [7,8]. A previous study conducted in 2002 in Quebec found that about one fifth of individuals who initiated an antidiabetic medication used a combination of antihypertensive, lipid-lowering and antiplatelet agents [9]. Men and those with a history of cardiovascular disease were more likely to use cardioprotective medications [9]. 

Considering the role medications can play in health outcomes, it appears important to describe the medications that are used by individuals with diabetes. We therefore aimed to (1) describe the number of medications and types of pharmacological classes used by older individuals with diabetes; (2) calculate the proportion of individuals using the combination of antidiabetic, antihypertensive and lipid-lowering agents, and (3) identify factors associated with using this combination.

## 2. Materials and Methods 

### 2.1. Data Source

We used the Québec Integrated Chronic Disease Surveillance System (QICDSS) from the Institut National de Santé Publique du Québec (INSPQ). The QICDSS combines five health administrative databases from the public health system. Using an encrypted unique health insurance number, it thereby links data from the health insurance registry, the hospital discharge database (MED-ECHO), the death registry, the fee-for-service physician claims database, and the pharmaceutical services [10]. The data included in the pharmaceutical services file come from the Public Prescription Drug Insurance Plan, which covers nearly 90% of individuals aged ≥65 years in Quebec [10]. The file contains information related to medication claims, including the date of delivery, the medication common denomination (chemical name of the compound), the DIN, the dosage, the number of days of treatment, the route of administration, and the prescriber.

### 2.2. Study Population

We created a population-based cohort study from the QICDSS by including individuals with prevalent diabetes aged between 66 and 75 years from April 1st, 2014 to March 31st, 2015. The upper age cut-off of 75 years was chosen to ensure that the individuals were most likely to benefit from the use of preventive medications (i.e., life expectancy >10 years) [4]. In order to draw a complete portrait of medication use, all individuals had to be covered by the universal Public Prescription Drug Insurance Plan for the entire 2014–2015 fiscal year or until their death, if applicable. Individuals in public long-term care facilities were excluded because their pharmacological data were not available. We used a validated definition of diabetes to identify the individuals to be included in the cohort [11,12]. The algorithm of this case definition (2 physician claims within 2 years or 1 hospitalization with a diabetes diagnosis) is currently used across Canada for surveillance purposes and shows a very good accuracy (94.6% sensitivity and 87.9% PPV) [11,12]. The individuals meeting the case definition at any time between January 1st, 1996 and March 30th, 2013 were included in the cohort. A gap of at least one year between identification of cases of diabetes (prior to April 2013) and the actual inclusion in the cohort (April 2014) was used to ensure all individuals had at least one year of follow-up for diabetes-related medications to be prescribed.

### 2.3. Definition of Medication Use and Combination of Treatments

The individuals were deemed to have used a medication if there was a claim for the specific medication within the 2014–2015 fiscal year. We used the common denomination (each active ingredient or combination has a distinct common denomination code) to identify each medication used at the individual level and added them together to obtain the total number of medications used. We then regrouped the medications under classes using the American Hospital Formulary Service (AHFS) classification, which allowed us to describe the pharmacological profile for the whole cohort. 

In Canada, Clinical Practice Guidelines [4] recommend the use of antidiabetic agents, ACE inhibitors/ARBs and statins for older individuals with diabetes. As there is no clinical information in administrative databases, such as contra-indications to specific medication therapies, we extended the definitions of cardioprotective agents to include all antihypertensives (rather than ACEI/ARBs alone) and lipid-lowering agents (rather than statins alone). An individual was considered to be exposed to a combination of treatments as long as there was at least one claim for each of the three medication classes (antidiabetic, antihypertensive, lipid-lowering drugs) during the fiscal year. Consequently, all other possibilities with two or fewer of the agents mentioned above were considered as not exposed to the combination. 

### 2.4. Potential Factors Associated with the Combination of Treatments

Based on prior studies, we examined the influence of sociodemographic variables such as age, sex, and living area (by postal code), as well as the number of different medications used annually, on the likelihood of being exposed to the combination of treatments. By definition, people with cardiovascular disease may be more likely to receive the combination of medications because the combination includes cardiovascular medications. Therefore, we investigated the impact of cardiovascular comorbidities distinct from other comorbidities. We included coronary diseases and stroke in the group of cardiovascular comorbidities. The group of other comorbidities included six other diseases with validated definitions in the QICDSS [10]: chronic obstructive pulmonary disease (COPD), asthma, anxio-depressive disorders, schizophrenia, Alzheimer’s disease and related dementias, and osteoporosis. We also assessed the influence of material and social deprivation with the validated index developed by the INSPQ [13]. This ecological proxy is based on the individual’s postal code and information provided by the Canadian Census of Population conducted every five years by Statistics Canada. Data from the Census is applied to the area where the individual lives. The material deprivation index considers information on income, education and employment status to reflect the lack of everyday goods and commodities. The social deprivation index considers the proportion of people living alone, lone-parent families and people who are divorced, separated or widowed. It refers to the fragility of the individual’s social network. Both deprivation indices are presented in quintile, where quintile 1 represents the most privileged, while quintile 5 represents the most deprived individuals. 

### 2.5. Statistical Analysis

Descriptive statistics, including mean, standard deviation and proportions, were used to describe the cohort and the pharmacological profile. Student’s t-test was performed to assess if there were statistically significant differences in the mean number of medications used between the individuals exposed or not exposed to the combination of treatments. 

We used multivariable Poisson regression analysis with robust error variance to model and identify factors associated with the combination of treatments. The Poisson regression model with robust error estimate of the variance allows for the estimation of relative risk without variance overestimation [14]. We created two models: one including all variables and another without the number of medications. We proceeded this way because the number of medications is potentially an intermediate variable, as it may intervene between some independent variables and exposure to the combination of treatments. The inclusion of this variable can thus falsely conceal or modify the effect of other independent variables when included in the regression model [15]. 

All of the analyses were performed using SAS© version 9.3. Statistical tests, which were 2-sided with significance levels defined at 0.01.

### 2.6. Sensitivity Analysis

In a sensitivity analysis, we considered an alternative definition of the combination of treatments, by requiring an antiplatelet agent in addition to the three other medication classes. An antiplatelet agent is recommended when a person has cardiovascular risk factors or has a history of cardiovascular disease [4]. 

### 2.7. Ethics

The QICDSS was created according to stringent standards of security and privacy. The use of the QICDSS for surveillance has been approved by the government bodies that own the databases, the Public Health Ethics Committee and the Commission d’Accès à l’Information du Québec. The present study meets all the ethics requirements related to the use of the QICDSS data for surveillance purposes and, therefore, neither specific research ethics approval nor individual participant consent were required for this project in accordance with the Canadian Tri-Council Policy Statement: Ethical Conduct for Research Involving Humans. 

## 3. Results

### 3.1. Characterictics

There were 146,710 individuals with diabetes who met the inclusion criteria. As shown in Table 1, the mean age was 70.6 years (±2.7) and 55.8% were men. On average, the individuals had one of the eight comorbidities (1.1 ± 1.1) (data not shown) and used 11.8 (±6.7) different medications annually. A small percentage (2.8%) did not use any medication (data not shown). The proportion of individuals using ≥5 medications was 89.8%, and 58.8% used ≥10 medications. 

### 3.2. Medication Use According the AHFS Classification

The proportion of users for each AHFS medication class is shown in Table 2. The three most frequent medication classes were cardiovascular (91.3%), which include antihypertensive agents and lipid lowering agents; hormones and synthetic substitutes (84.5%), which include antidiabetic agents; and central nervous system agents (79.8%), including analgesics, benzodiazepines, antidepressants, and antipsychotics. Around half of the cohort (47.2%) also used gastro-intestinal medications.

### 3.3. Combination of Treatment

A proportion of 58.6% of the population was exposed to the combination of treatments (Table 1). The prevalence reached 60.9% in men and 55.7% in women (Table 3). More specifically, the proportion of individuals who used antidiabetic agents was 75.9%; 83.1% used antihypertensive agents; 78.9% used lipid-lowering agents; and 58.1% used antiplatelet agents. 

The factors most strongly associated with the combination of treatments included the presence of cardiovascular comorbidities (RR = 1.29 [99% CI: 1.28–1.31]), and material deprivation quintiles higher than 1 (from 1.06 [1.04–1.07] for quintile 2 to 1.09 [1.07–1.11] for quintile 4) (Table 3, Model 1). Other factors that were weakly associated with the combination of treatments included male sex (1.04 [1.03–1.05]) and the 71–75 years age group (1.02 [1.01–1.03]). The presence of comorbidities other than cardiovascular diseases (0.92 [0.91–0.93]) was associated with a lower probability of being exposed to the combination of treatments. When included in the model, the number of medications was the strongest factor associated with using the combination of treatments (Table 3, Model 2). The mean number of medications used annually was indeed higher among those exposed to the combination of treatments than among those who were not (13.8 ± 6.3 vs 9.1 ± 6.3 p < 0.0001).

### 3.4. Sensitivity Analysis

The sensitivity analysis showed that the proportion of individuals exposed to the combination of treatments decreased from 58.6% to 41.5% when using the alternative definition including antiplatelet agents (Appendix A, Table A1). The factors associated with the use of the combination of treatments remained similar with the alternative definition.

## 4. Discussion

In this population-based study, we found that older people with diabetes use around 12 different medications per year, and that nearly 60% are exposed to a combination of antidiabetic and cardioprotective treatments. A high number of medications, the presence of cardiovascular comorbidities, the absence of comorbidities other than cardiovascular, high material and social deprivation, and male sex were associated with the combination of treatments. 

Individuals included in the cohort consumed a large number of medications. Close to 90% of the individuals used ≥5 different medications and 59% used ≥10. In comparison, 75% of individuals over 65 years used ≥5 medications in 2014–2015, while 39% used at least 10 in Quebec [16]. Diabetes is known to be an important factor leading to polypharmacy [17], although temporality cannot be ascertained in our study as we used prevalence data. In addition to antidiabetic medications, polypharmacy frequently included cardiovascular medications, central nervous system medications (e.g., benzodiazepines, antidepressants) and proton pump inhibitors. With a minimum of three medications needed to manage diabetes and cardiovascular risk factors, it is understandable that most individuals in our study used ≥5 medications. Nonetheless, this large number of medications may lead to adverse drug events and interactions, and a higher mortality rate [18]. It is important to focus on priority medications, while reducing questionable medications such as potentially inappropriate medications [19].

Three quarters (76%) of the individuals in our cohort used an antidiabetic agent. This proportion is lower than for the whole Canadian population ≥60 years, that reached 86% in 2009–2010 [20]. In the United States, around 14% of insured adults with diabetes do not use medication to manage glycemia [21]. Individuals ≥65 years are less likely to be treated with diet only [21]. Over time, it becomes difficult to achieve the glycemic targets without medication [22]. Hence, a significant proportion of the individuals without antidiabetic agents in our study may have been undertreated and could have benefited from therapy. This high proportion of non-users may result from a delay between the time of diagnosis and the initiation of pharmacological treatment or because of a lack of adherence to treatment. Nonetheless, some of these individuals might also be false positive diabetes cases, although it may be difficult to estimate how many they represent.

The use of antihypertensive and lipid-lowering agents was high in the study. A proportion of 83% of the individuals used an antihypertensive agent, while 79% used a lipid-lowering agent. As statins were the most frequently prescribed medication in 2016 in Canada, it is not surprising to find such a high proportion of users [23]. This may result from the increased awareness of the consequences of high blood pressure and high cholesterol levels on cardiovascular outcomes over the years.

About half of the individuals in our cohort were using an antiplatelet agent. This result can be explained by the change of clinical practice guidelines towards an optional treatment, and the bleeding risks associated with use of antiplatelet agents in older individuals [4]. The individuals with previous cardiovascular disease were more likely to have an antiplatelet agent, which is consistent with recommendations for secondary prevention. Furthermore, as aspirin is available over the counter, databases may underestimate its actual use, although few older individuals use it without prescription in Quebec [24].

Factors associated with the combination of treatments included male sex and the presence of cardiovascular disease. These factors are similar to the ones identified in 2002 among new users of antidiabetic agents in older Quebecers [9]. Conversely, in Saskatchewan, Canada, there were very few differences between males and females among individuals aged ≥55 years with respect to cardioprotective medications (without antiplatelet agents) [25]. Other studies have observed that treatments for diabetes and cardiovascular risk factors are suboptimal for older individuals with diabetes [26,27]. Considering 63% of general practitioners in Quebec report using diabetes guidelines [28], increasing practitioners’ awareness may have a significant impact on the proportion of patients benefiting from optimal follow-up and pharmacological treatment. Nonetheless, recommendations in guidelines are not based exclusively on high-quality evidence [29]. Therefore, decisions on treatment could also be influenced by the patient’s risk factor levels, values and preferences, among other elements. For example, the combination of treatments may not be suitable for individuals whose glycemia or cardiovascular risk factors are well controlled with lifestyle, and for those for whom the risk/benefit ratio of using preventive medication is high (e.g. life expectancy <10 years due to diseases). The administrative databases do not allow for the identification of those cases. Moreover, our study design did not allow for the identification of other potential reasons for not initiating treatments, such as intolerability, costs, patient values and preferences, clinician values and preferences or overall concern for over medication. Considering these conditions, the fact that more than 75% of all older adults with diabetes are exposed to each cardioprotective medication appears encouraging at a population level. 

This study has limitations mainly related to the fact that it is an observational study performed with administrative databases. Firstly, although we used a validated definition of diabetes, some false positive cases might have been included in the cohort. Secondly, without clinical data on lifestyle, severity of diabetes, risk factors control, and attainment of clinical targets, it was not possible to determine the clinical necessity of the combination of treatments. Nonetheless, we ensured that the life expectancy in general was sufficient to obtain benefits from preventive medications by only including people aged 66–75 years. Since the definition of medication use required only one claim, it did not allow for the evaluation of treatment adherence that is necessary to benefit from therapy. Likewise, we did not study the health outcomes to evaluate the impact of the combination of treatments. Finally, the database is composed of medication claims and not prescriptions. Some patients may decide not to initiate treatment, which may underestimate prescribers’ intent to treat. 

## 5. Conclusions

Older individuals with diabetes use a large number of medications. The most commonly used medication classes included cardiovascular drugs and hormone agents, as expected. However, the high prevalence of central nervous system and digestive system medications may raise questions about the overall quality of pharmacotherapy and the potential associated risks. Almost 60% of the individuals received a combination of antidiabetic, lipid-lowering and antihypertensive medications, yet the prevalence of each medication class exceeded 75%. At a population level, this use is encouraging. There is a need to determine if such exposure also translates into cardiovascular benefits at the population level. The impact of polypharmacy, which carries a potentially significant iatrogenic risk, should also be considered.

## Figures and Tables

**Table 1:** Characteristics of the individuals included in the study according to the use of the combination * of treatments.

	Total	Exposed to the Combination of Treatments	Not Exposed to the Combination of Treatments
**N (%)**	146,710 (100.0%)	85,977 (58.6%)	60,733 (41.4%)
**Sex**			
Female	64,794 (44.2%)	36,079 (42.0%)	28,715 (47.3%)
Male	81,916 (55.8%)	49,898 (58.0%)	32,018 (52.7%)
**Age**			
**Mean (years) (SD)**	70.6 (2.7)	70.6 (2.7)	70.5 (2.7)
66–70 years	73,507 (50.1%)	42,455 (49.4%)	31,052 (51.1%)
71–75 years	73,203 (49.9%)	43,522 (50.6%)	29,681 (48.9%)
**Material Deprivation Quintile**			
1 (most privileged)	20,515 (14.0%)	11,360 (13.2%)	9155 (15.1%)
2	25,243 (17.2%)	14,751 (17.2%)	10,492 (17.3%)
3	27,674 (18.9%)	16,499 (19.2%)	11,175 (18.4%)
4	31,550 (21.5%)	18,996 (22.1%)	12,554 (20.7%)
5 (most deprived)	32,909 (22.4%)	19,325 (22.5%)	13,584 (22.4%)
Missing	8819 (6.0%)	5046 (5.9%)	3773 (6.2%)
**Social Deprivation Quintile**			
1 (most advantaged)	24,670 (16.8%)	14,161 (16.5%)	10,509 (17.3%)
2	26,548 (18.1%)	15,656 (18.2%)	10,892 (17.9%)
3	28,585 (19.5%)	16,848 (19.6%)	11,737 (19.3%)
4	28,516 (19.4%)	16,694 (19.4%)	11,822 (19.5%)
5 (most deprived)	29,572 (20.2%)	17,572 (20.4%)	12,000 (19.8%)
Missing	8819 (6.0%)	5046 (5.9%)	3773 (6.2%)
**Cardiovascular Comorbidities**			
No	85,989 (58.6%)	44,990 (52.3%)	40,999 (67.5%)
Yes	60,721 (41.4%)	40,987 (47.7%)	19,734 (32.5%)
**Other Comorbidities**			
No	82,906 (56.5%)	49,785 (57.9%)	33,121 (54.5%)
Yes	63,804 (43.5%)	36,192 (42.1%)	27,612 (45.5%)
**Living Area**			
Urban	113,044 (77.1%)	66,128 (76.9%)	46,916 (77.3%)
Rural	33,230 (22.7%)	19,622 (22.8%)	13,608 (22.4%)
Missing	436 (0.3%)	227 (0.3%)	209 (0.3%)
**Yearly Number of Medications**			
**Mean (SD)**	11.8 (6.7)	13.8 (6.3)	9.1 (6.3)
0 to 4	15,030 (10.2%)	1037 (1.2%)	13,993 (23.0%)
5 to 9	45,476 (31.0%)	22,775 (26.5%)	22,701 (37.4%)
10 to 14	43,680 (29.8%)	29,933 (34.8%)	13,747 (22.6%)
15 to 19	24,060 (16.4%)	17,960 (20.9%)	6100 (10.0%)
20 and more	18,464 (12.6%)	14,272 (16.6%)	4192 (6.9%)

* The combination of treatments includes at least one of each of the following drug classes: antihypertensive, antidiabetic, and lipid-lowering agents. SD: Standard deviation.

**Table 2:** Prevalence of medication use according to the AHFS Classification.

AHFS Classification	Number of Users (%)
Cardiovascular (24.00)	133,926 (91.3%)
Hormones (68.00)	123,968 (84.5%)
Central nervous system (28.00)	117,086 (79.8%)
Gastrointestinal (56.00)	69,229 (47.2%)
Electrolytic, caloric and water balance (40.00)	68,270 (46.5%)
Anti-infective (8.00)	55,856 (38.1%)
Autonomic (12.00)	48,271 (32.9%)
Dietary supplements (88.00)	40,372 (27.5%)
Skin and mucous membrane preparations (84.00)	40,371 (27.5%)
Ophtalmic, otic and nasal ointments solutions suspensions and preparations (52.00)	36,849 (25.1%)
Blood formation and coagulation (20.00)	36,260 (24.7%)
Other (92.00)	31,572 (21.5%)
Smooth muscle relaxants (86.00)	6403 (4.4%)
Antineoplastic (10.00)	4945 (3.4%)
Expectorants and cough preparations (48.00)	2882 (2.0%)
Antihistamines (4.00)	419 (0.3%)

**Table 3:** Prevalence and relative risk of being exposed to the combination* of treatments.

	n/N (%)	Crude RR (99% CI)	Adjusted RR (99% CI) Model 1 **	Adjusted RR (99% Cl) Model 2 ***
**Sex**				
Female	36,079/64,794 (55.7%)	1	1	1
Male	49,898/81,916 (60.9%)	1.09 (1.08–1.10)	1.04 (1.03–1.05)	1.12 (1.11–1.13)
**Age**				
66–70 years	42,455/73,507 (57.8%)	1	1	1
71–75 years	43,522/73,203 (59.5%)	1.03 (1.02–1.04)	1.02 (1.01–1.03)	0.98 (0.98–0.99)
**Material Deprivation Quintile**				
1 (most privileged)	11,360/20,515 (55.4%)	1	1	1
2	14,751/25,243 (58.4%)	1.06 (1.04–1.07)	1.06 (1.04–1.07)	1.03 (1.02–1.05)
3	16,499/27,674 (59.6%)	1.08 (1.06–1.09)	1.08 (1.06–1.09)	1.04 (1.03–1.06)
4	18,996/31,550 (60.2%)	1.09 (1.07–1.10)	1.09 (1.07–1.11)	1.05 (1.04–1.06)
5 (most deprived)	19,325/32,909 (58.7%)	1.06 (1.04–1.08)	1.06 (1.05–1.08)	1.04 (1.02–1.05)
Missing	5046/8819 (57.2%)	1.03 (1.01–1.06)	1.06 (1.03–1.08)	1.01 (0.99–1.03)
**Social Deprivation Quintile**				
1 (most privileged)	14,161/24,670 (57.4%)	1	1	1
2	15,656/26,548 (59.0%)	1.03 (1.01–1.04)	1.03 (1.01–1.04)	1.01 (0.99–1.02)
3	16,848/28,585 (58.9%)	1.03 (1.01–1.04)	1.03 (1.01–1.04)	1.00 (0.99–1.01)
4	16,694/28,516 (58.5%)	1.02 (1.01–1.03)	1.02 (1.01–1.04)	1.00 (0.99–1.01)
5 (most deprived)	17,572/29,572 (59.42%)	1.04 (1.02–1.05)	1.04 (1.02–1.05)	1.00 (0.98–1.00)
missing	5046/8819 (57.2%)	1.00 (1.00–1.02)	1.06 (1.03–1.08)	1.01 (0.99–1.03)
**Cardiovascular Comorbidities**				
No	44,990/85,989 (52.3%)	1	1	1
Yes	40,987/60,721 (67.5%)	1.29 (1.28–1.30)	1.29 (1.28–1.31)	1.08 (1.07–1.09)
**Other Comorbidities**				
No	49,785/82,906 (60.1%)	1	1	1
Yes	36,192/63,804 (56.7%)	0.94 (0.94–0.95)	0.92 (0.91–0.92)	0.81 (0.80–0.82)
**Living Area**				
Urban	66,128/113,044 (58.5%)	1	1	1
Rural	19,622/33,230 (59.1%)	1.01 (1.00–1.02)	0.99 (0.98–1.00)	0.98 (0.97–0.99)
Missing	227/436 (52.1%)	0.98 (0.89–1.08)	0.97 (0.88–1.07)	0.95 (0.86–1.03)
**Number of Medications**				
0 to 4	1037/15,030 (6.9%)	1		1
5 to 9	22,775/45,476 (50.1%)	7.26 (6.84–7.70)		7.26 (6.84–7.71)
10 to 14	29,933/43,680 (68.5%)	9.93 (9.36–10.54)		10.15 (9.57–10.76)
15 to 19	17,960/24,060 (74.7%)	10.82 (10.20–11.48)		11.33 (10.68–12.02)
20 and more	14,272/18,464 (77.3%)	11.20 (10.56–11.89)		12.13 (11.43–12.87)

* The combination of treatments includes at least one of each of the following drug classes: antihypertensive, antidiabetic, and lipid-lowering agents; RR: Rate ratio; CI: Confidence interval; ** Model 1 includes: sex, age, material deprivation quintiles, social deprivation quintiles, cardiovascular comorbidities, other comorbidities, and living area.; *** Model 2 includes: sex, age, material deprivation quintiles, social deprivation quintiles, cardiovascular comorbidities, other comorbidities, living area, and number of medications.

## Appendix A

**Table A1:** Prevalence and relative risks of being exposed to the combination * of treatments, including antiplatelet agents (sensitivity analysis).

	n/N (%)	Crude RR (99% CI)	Adjusted RR (99% CI) Model 1 **	Adjusted RR (99% Cl) Model 2 ***
**Sex**				
Female	23,630/64,794 (36.5%)	1	1	1
Male	37,186/81,916 (45.4%)	1.24 (1.23–1.26)	1.14 (1.13–1.15)	1.24 (1.22–1.25)
**Age**				
66–70 years	29,750/73,507 (40.5%)	1	1	1
71–75 years	31,066/73,203 (42.3%)	1.05 (1.04–1.06)	1.02 (1.01–1.03)	0.98 (0.97–0.99)
**Material Deprivation Quintile**				
1 (most privileged)	7741/20,515 (37.7%)	1	1	1
2	10,452/25,243 (41.4%)	1.10 (1.07–1.12)	1.09 (1.07–1.12)	1.06 (1.04–1.09)
3	11,565/27,674 (41.8%)	1.11 (1.08–1.13)	1.10 (1.08–1.12)	1.06 (1.04–1.08)
4	13,644/31,550 (43.3%)	1.15 (1.12–1.17)	1.14 (1.11–1.16)	1.09 (1.07–1.11)
5 (most deprived)	13,811/32,909 (42.0%)	1.11 (1.09–1.14)	1.10 (1.08–1.13)	1.07 (1.04–1.09)
Missing	3603/8819 (40.9%)	1.08 (1.05–1.12)	1.12 (1.08–1.16)	1.05 (1.02–1.08)
**Social Deprivation Quintile**				
1 (most privileged)	9853/24,670 (39.9%)	1	1	1
2	11,023/26,548 (41.5%)	1.04 (1.02–1.06)	1.03 (1.01–1.06)	1.01 (0.99–1.03)
3	11,977/28,585 (41.9%)	1.05 (1.03–1.07)	1.05 (1.03–1.07)	1.02 (1.00–1.04)
4	11,778/28,516 (41.3%)	1.03 (1.01–1.06)	1.05 (1.03–1.07)	1.02 (1.00–1.04)
5 (most deprived)	12,582/29,572 (43.6%)	1.07 (1.04–1.09)	1.07 (1.05–1.10)	1.02 (1.00–1.04)
Missing	3603/8819 (40.9%)	1.02 (0.99–1.05)	1.12 (1.08–1.16)	1.05 (1.02–1.08)
**Cardiovascular Comorbidities**				
No	27,177/85,989 (31.6%)	1	1	1
Yes	33,639/60,721 (55.4%)	1.75 (1.73–1.77)	1.74 (1.72–1.76)	1.39 (1.37–1.40)
**Other Comorbidities**				
No	35,061/82,906 (42.3%)	1	1	1
Yes	25,755/63,804 (40.4%)	0.95 (0.94–0.97)	0.90 (0.89–0.91)	0.77 (0.76–0.78)
**Living Area**				
Urban	46,317/113,044 (41.0%)			
Rural	14,343/33,230 (43.2%)	1.05 (1.04–1.07)	1.03 (1.01–1.04)	1.01 (1.00–1.03)
Missing	156/436 (35.8%)	1.01 (0.89–1.15)	0.97 (0.85–1.10)	0.94 (0.83–1.06)
**Number of Medications**				
0 to 4	227/15,030 (1.5%)	1		1
5 to 9	13,865/45,476 (30.5%)	20.19 (17.73–22.99)		19.78 (17.37–22.51)
10 to 14	21,649/43,680 (49.6%)	32.82 (28.83–37.35)		31.95 (28.07–36.36)
15 to 19	13,797/24,060 (57.3%)	37.97 (33.35–43.22)		37.02 (32.53–42.14)
20 and more	11,278/18,464 (61.1%)	40.44 (35.53–46.04)		39.86 (35.01–45.39)

* The combination of treatments includes at least one of each of the following drug classes: antihypertensive, antidiabetic, lipid-lowering, and antiplatelet agents; RR: Rate ratio; CI: Confidence interval; ** Model 1 includes: sex, age, material deprivation quintiles, social deprivation quintiles, cardiovascular comorbidities, other comorbidities, and living area; *** Model 2 includes: sex, age, material deprivation quintiles, social deprivation quintiles, cardiovascular comorbidities, other comorbidities, living area, and number of medications.
